# Factors controlling the transfer of biogenic organic species from seawater to sea spray aerosol

**DOI:** 10.1038/s41598-022-07335-9

**Published:** 2022-03-04

**Authors:** Mitchell V. Santander, Jamie M. Schiffer, Christopher Lee, Jessica L. Axson, Michael J. Tauber, Kimberly A. Prather

**Affiliations:** 1 Department of Chemistry and Biochemistry, University of California, San Diego, La Jolla, CA 92093 USA; 2 Takeda, San Diego, CA 92121 USA; 3 Scripps Institution of Oceanography, University of California, San Diego, La Jolla, CA 92037 USA; 4 Bloomfield Hills, MI USA

**Keywords:** Biogeochemistry, Marine chemistry

## Abstract

Ocean waves transfer sea spray aerosol (SSA) to the atmosphere, and these SSA particles can be enriched in organic matter relative to salts compared to seawater ratios. A fundamental understanding of the factors controlling the transfer of biogenic organic matter from the ocean to the atmosphere remains elusive. Field studies that focus on understanding the connection between organic species in seawater and SSA are complicated by the numerous processes and sources affecting the composition of aerosols in the marine environment. Here, an isolated ocean–atmosphere system enables direct measurements of the sea–air transfer of different classes of biogenic organic matter over the course of two phytoplankton blooms. By measuring excitation–emission matrices of bulk seawater, the sea surface microlayer, and SSA, we investigate time series of the transfer of fluorescent species including chlorophyll-a, protein-like substances, and humic-like substances. Herein, we show the emergence of different molecular classes in SSA at specific times over the course of a phytoplankton bloom, suggesting that SSA chemical composition changes over time in response to changing ocean biological conditions. We compare the temporal behaviors for the transfer of each component, and discuss the factors contributing to differences in transfer between phases.

## Introduction

Breaking waves in the ocean produce sea spray aerosols (SSA), and the ocean represents a dominant source of atmospheric aerosols^1–4^. SSA particles are comprised of a complex array of biogenic species, including intact microbes, which control the physicochemical properties of SSA and thus affect their atmospheric behavior^3^. In recent decades, significant effort has been aimed at trying to establish a link between the composition of SSA and the biological state of the ocean^5–9^, specifically related to phytoplankton growth^10–14^ and the microbial loop^5,15–17^.

In addition to biological processes, physical and chemical processes play a complex role in affecting the composition of the atmosphere. Physical factors such as changes in SSA flux or the physical size of molecules and particles in seawater determine the efficiency of sea–air transfer of organic matter^18,19^. Previous studies have suggested that increased dissolved organic carbon (DOC) concentrations or increased surface activity of the chemical species of interest enhance the transfer process^15,20–22^. Many hydrophobic biogenic species have been shown to be enriched in the sea-surface microlayer (SSML)^23–26^, the uppermost layer (1–1000 μm) on top of the ocean that serves as a critical environmental interface^27,28^. However, observed associations between specific classes of organic molecules, phytoplankton growth, and seawater-SSML-SSA transfer have been inconsistent between multiple studies.

Here, we investigate the physical and chemical mechanisms controlling the sea–air transfer of organic species in SSA by probing the composition of collected bulk seawater, SSML, and SSA samples with excitation-emission matrix (EEM) spectroscopy in combination with parallel factor analysis (PARAFAC)^29–31^. EEM spectroscopy is an extension of fluorescence spectroscopy that has been increasingly used to characterize organic matter in a variety of aquatic systems, including seawater^29,32,33^. Using EEMs for chemical characterization allows for direct, rapid measurements^32^ and minimizes the possibility of artifacts created by sample storage or harsh processing conditions. Three sets of biologically diverse fluorophores are monitored: (1) chlorophyll-a, which is an indicator for phytoplankton biomass; (2) protein-like substances; and (3) humic-like substances (HULIS)^29–31^. Additionally, we use PARAFAC analysis to separate EEMs into a set of individual fluorescent components^29,33–35^. Changes in fluorescence signatures over time are used to provide insight into the factors affecting the transfer of biogenic organic species from the ocean to the atmosphere.

## Methods

### Phytoplankton bloom experiments

Two phytoplankton blooms were induced in an indoor tank. The first phytoplankton bloom experiment (MART A) was conducted in January 2014 and lasted 17 days, while the second experiment (MART B) took place in April 2014 and lasted 26 days. Both microcosm experiments were induced using methods described previously^15^. In these experiments, 60 L of seawater from the Scripps Institution of Oceanography pier was filtered using a 50 μm mesh screen to remove debris and zooplankton that graze on phytoplankton (Sefar Nitex 03-100/32) and placed into a Marine Aerosol Reference Tank (MART)^36^. Seawater conditions at the time of sampling are included in the Supplemental Information (Table S2). Seawater was spiked with Guillard’s F medium (diluted by a factor of 2) to induce a phytoplankton bloom^37^. Two fluorescent tubes (Full Spectrum Solutions, model 205457; T8 format, color temperature 5700 K, 2950 lumens) were attached to the sides of the MART to promote the growth of phytoplankton^38^. The low light levels relative to solar conditions^15^, the absence of UV radiation, and the relatively short lifetime of SSA in the MART (minutes)^36^ makes it unlikely that photochemistry would cause differences between the SSA and seawater (bulk, or SSML). Similar lighting for both tanks also rules out photochemical change as an origin for any differences between MART A and MART B microcosms. The progress of the phytoplankton bloom was monitored daily by measuring in vivo chlorophyll-a fluorescence using a handheld fluorimeter (Turner Designs, Aquafluor). The handheld fluorimeter measures the fluorescence at 395 nm excitation and ≥ 660 nm emission.

### Generation of aerosols and sample collection

The MART uses a periodic plunging waterfall to generate aerosols, a method that has been described previously^15,36^. The MART consists of an isolated system that allows for the study of SSA without the influence of aerosol particles from other non-marine sources. While physical processes such as wind are not reproduced in the MART system, the MART plunging waterfall has been shown to produce a bubble size distribution that is similar to the bubble size distribution observed in the ocean. Thus, the MART produces SSA size distributions that mimic the size distributions of nascent SSA over the ocean, allowing us to solely focus on the particles produced during the bubble bursting process. Aerosol generation occurred for two hours followed by two hours of no particle generation or seawater mixing. Aerosols were collected daily using glass impingers (Chemglass, CG-1820, 0.2 μm D_p_ lower cutoff at 1 LPM) loaded with 20 mL of ultrapure (Type 1) water. The airflow through the impingers was 1 LPM for 2 h. The impingers were cleaned daily by heating to 500 °C for 7 h. In MART B, three impingers were used in parallel to collect SSA.

All bulk seawater and SSML samples were collected in vials that were previously cleaned with an acid-rinse using 0.1 N HCl followed by multiple rinses of ultrapure water, and then heating at 500 °C for 7 h. Bulk seawater samples were collected daily from a spigot located on the side of the MART. SSML samples were collected using the glass plate method once every two days to prevent excessive depletion of the microlayer^28^. The SSML was collected in between wave-breaking periods. During this time, the MART waterfall is turned off and no bubbles are produced. SSML collection occurs ~ 10–15 min after waterfall and bubble production stops. Previous studies report that the SSML re-establishes itself within seconds^26^ and is thus established prior to sample collection. Organic and biological material scavenged and brought to the surface during the prior wave-breaking periods remains at the surface and is collected via the glass plate method. The glass plate was cleaned directly before and after each collection by rinsing with 1 N HCl, followed by an ethanol rinse, and multiple rinses using ultrapure water.

To examine the roles of phytoplankton and bacteria, both bulk seawater and SSML were filtered using a microanalysis vacuum filter setup (EMD Millipore Corp, Cat. Num. XA5002501) and hand pump (Fisher Scientific, Part Num. 1367811E). Samples were first analyzed without filtering, and then filtered sequentially (both size filters used were 25 mm polycarbonate Isopore Membrane filter) with a 2.0 μm filter (EMD Millipore Corp, GTTP02500) to remove large phytoplankton^39^ and again with a 0.2 μm filter (EMD Millipore Corp, TTTP02500) to remove bacteria, allowing viruses and dissolved organic carbon (DOC) to pass through^40^. EEMs were obtained after each filtration. All glassware used during filtration (including the stainless steel mesh support screen) was rinsed with ultrapure water and cleaned by heating to 500 °C for 7 h after use. Polycarbonate filters were sterilized using 1 N HCl and rinsed with ultrapure water.

### Excitation–emission matrices and parallel factor analysis

EEMs were obtained for all samples using a spectrofluorometer (Horiba Scientific, Aqualog with extended range). Three aliquots were taken from each of the three impingers, and an EEM was measured for each sample (total of nine EEMs per day for SSA phase). Excitation wavelengths ranged from 235 to 450 nm. Emission ranged from 250 to 800 nm. For the second half of the MART A experiment, EEM measurements were performed using a standard Aqualog spectrofluorometer covering an emission wavelength range of 250–620 nm. A background spectrum acquired with ultrapure water was subtracted from all EEMs. EEMs were corrected for inner-filter effects based on absorbance spectra measured simultaneously. Rayleigh scatter (1st and 2nd order) was removed in the analysis using an interpolation method^41^. EEMs were also normalized to the area of the Raman Scattering peak of water at 350 nm excitation to convert fluorescence intensities to Raman Units (R.U.)^42,43^. The EEMs converted to R.U. were incorporated into the PARAFAC models. EEMs included in figures were smoothed with a Savitzky-Golay filter (4th order, 15-point for MART A, 4th order, 9-point for MART B to prevent distorting the narrower chlorophyll-a peak)^44^. The smoothing of EEMs was not done prior to PARAFAC modeling or any other data analysis. All error bars shown, unless otherwise noted, represent the standard deviation of the mean (N = 9) for the 9 SSA measurements.

PARAFAC modeling for each of the two MARTs was carried out using the DOMFluor toolbox in MATLAB (The MathWorks, Inc.)^34,35^. The PARAFAC model for MART A consisted of 274 EEMs, while the MART B PARAFAC model used 451 EEMs. Both PARAFAC models’ datasets consisted of EEMs for all phases (bulk seawater, SSML, and SSA). In order to model the complete MART A dataset, which contains EEMs from two spectrofluorometers with different emission wavelength ranges and increments, the dataset EEMs were cut and linearly interpolated to give emission wavelengths that ranged from 250 to 600 in 2 nm increments. For both MART A and MART B, a PARAFAC model was validated using split-half validation as well as random initialization analysis to ensure that the model generated was not a local minimum but a least squares result^34^.

## Results and discussion

### Phytoplankton blooms in two laboratory-based microcosms

Two phytoplankton microcosm studies were carried out to study sea–air transfer that occurs as a function of bloom conditions. The two microcosm experiments were conducted in the MART and are referred to as MART A (seawater collected and experiment conducted in Jan. 2014) and MART B (collected/conducted in April, 2014). Seawater used in the MART A and MART B microcosm experiments had different initial chlorophyll-a levels (1.33 mg/m^3^ for MART A; 4.49 mg/m^3^ for MART B), and the same nutrient amounts were added to both MARTs (see “Methods”) to induce phytoplankton blooms.

**Figure 1:**
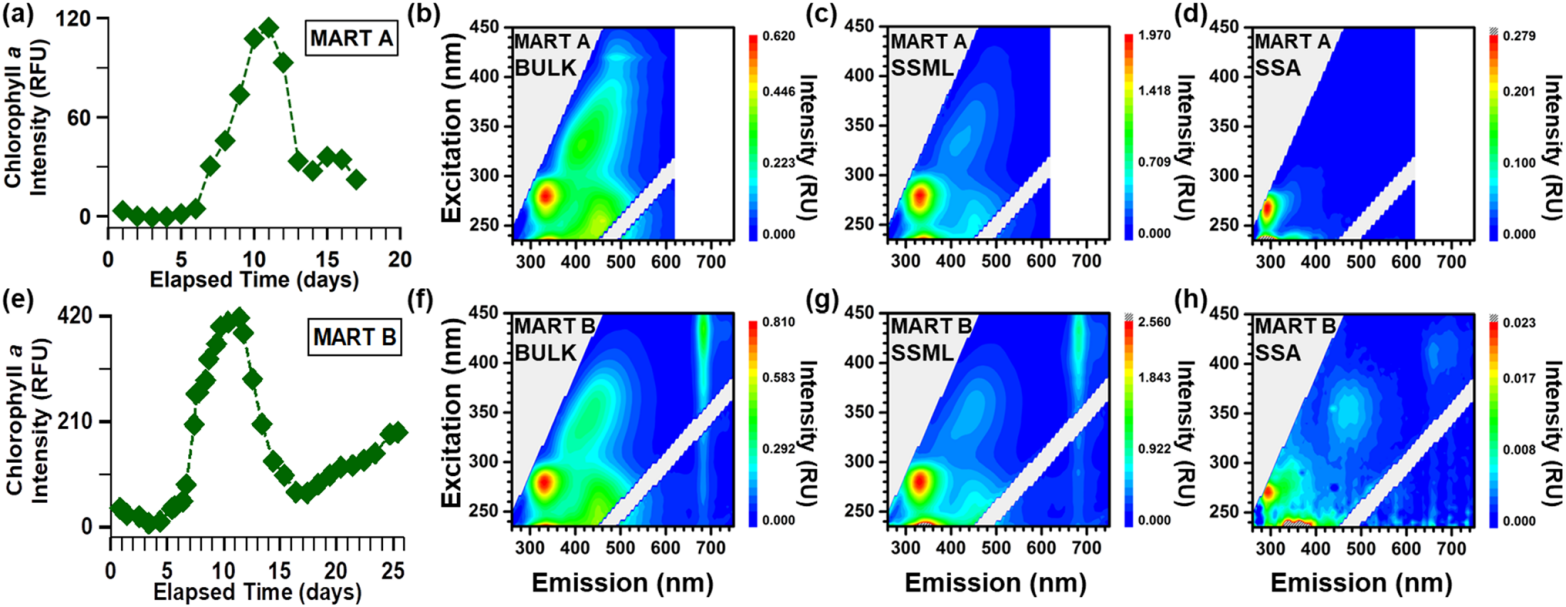
Chlorophyll-a time traces **(a,e)** and selected fluorescence excitation–emission matrices (EEMs) **(b–d,f–h)** for phytoplankton bloom experiments in MART A (top) and MART B (bottom). All EEMs shown are for unfiltered samples measured during the bloom decline stage (Day 17 for MART A, Day 15 for MART B). The EEMs shown are bulk seawater **(b,f)**, sea-surface microlayer (SSML) **(c,g)**, and sea spray aerosol (SSA) **(d,h)**. Dark gray regions (in UV excitation region of **g,h**) indicate amplitudes that exceed the given scale.

The progression of the phytoplankton bloom was monitored daily via in vivo chlorophyll-a fluorescence using a hand-held fluorometer (Fig. 1a,e). Based on these in vivo chlorophyll-a measurements, each bloom was categorized into three stages: (1) growth, which lasted 8–9 days for both blooms, (2) peak, which lasted for 3–4 days after the growth stage, and (3) decline, which persisted after the peak for 5 days in MART A and 14 days in MART B. We note that a second growth phase with lower chlorophyll-a levels developed in both microcosms. Although both MART microcosms were induced using the same nutrient additions, MART B reached a higher (~ 3 ×) chlorophyll-a concentration at the peak of the bloom (Fig. 1a,e). We investigate temporal changes in the transfer of different biogenic species transfer to SSA through these growth and death processes.

### Assignment of fluorescence bands and parallel factor analysis

In both phytoplankton bloom experiments, fluorescence was observed within EEM regions commonly detected in natural marine systems (Fig. 1b,c,f,g)^29,31^. The EEM fluorescence regions fall into three main classes: chlorophyll-a, protein-like substances, and HULIS. An example EEM depicting the three main fluorescence regions is included in the Supplemental Information (Fig. S1). The chlorophyll-a peak appeared at excitation/emission wavelengths of (400–440 nm)/(680–690 nm), while the HULIS peaks appeared at (260 or 360 nm)/(450–455 nm) and at (325 nm)/(410 nm)^29–31,45^. The peaks for protein-like substances were further divided into two regions: a tryptophan-like region at (< 235 and 275–280 nm)/(330–350 nm) and a tyrosine-like region at (< 235 and 275 nm)/(295–310 nm) for tyrosine-like species)^29,31^.

**Figure 2:**
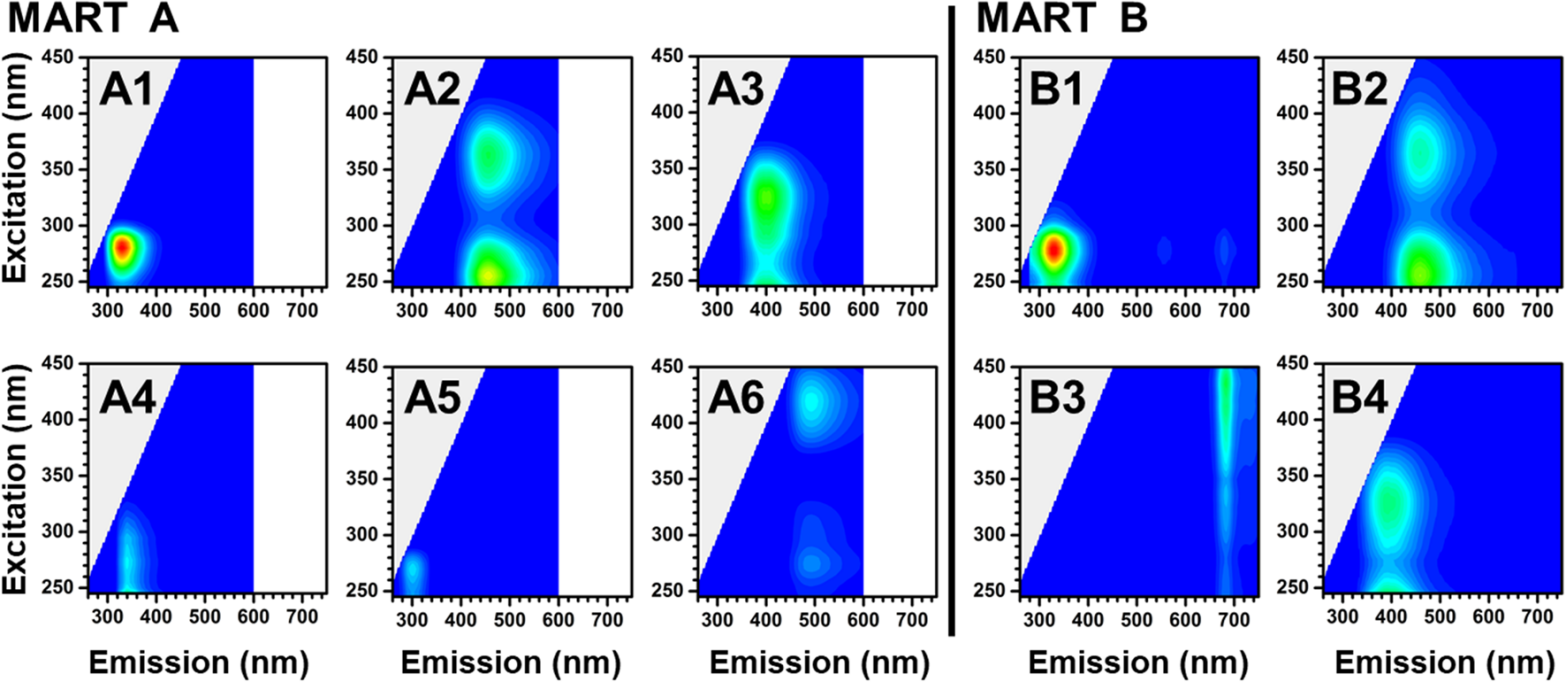
Representative components determined from PARAFAC analysis for MART A (A1–6, on left) and MART B (B1–4, on right). The components A1–6 have the same color scale as in Fig. 1b; the amplitudes are from PARAFAC decomposition of the EEM in that panel (MART A bulk seawater on day 17). Similarly, components B1–4 have the same color scale as in Fig. 1f, and the amplitudes result from decomposition of the EEM for MART B bulk seawater sample on day 15.

PARAFAC analysis was performed on the entire EEM dataset from each MART to distinguish the individual fluorophore contributions (Fig. 2, Table S1). Six components were found in MART A, and four components were found in MART B. PARAFAC components were associated with chromophores as follows: A1, A4, and B1 (tryptophan-like); A5 (tyrosine-like); B3 (chlorophyll-a); A2, A3, A6, B2, B4 (HULIS). Components A4, A5, and A6 only appeared in MART A (Table S1). Although a tyrosine-like feature (A5) was evident in the SSA EEMs for MART B, a tyrosine component was excluded from the PARAFAC model due to the small amplitude relative to other protein-like features and the number of SSA spectra within the dataset. Using the online OpenFluor spectral database^46^, all compounds identified here using a PARAFAC model were spectrally similar to components found in multiple marine environments^47–49^.

Within a single phase (bulk seawater, SSML or SSA), the fluorescence intensities of each component varied over time (Figs. S2, S3). It is important to note that we only compared the magnitude of fluorescence intensities within a single phase because of the different sampling methods used for bulk seawater, SSML and SSA. Thus, we focused on *temporal changes* in fluorescence intensity between phases. Moreover, we focused primarily on the temporal trends in MART B, which contained a more comprehensive SSA dataset. In the following sections, we describe how the fluorescence signals of chlorophyll-a, protein-like substances, and HULIS changed over the course of the phytoplankton bloom and discuss how these changes are associated with different factors influencing sea–air transfer.

### Chlorophyll-a in bulk seawater, SSML, and SSA

**Figure 3:**
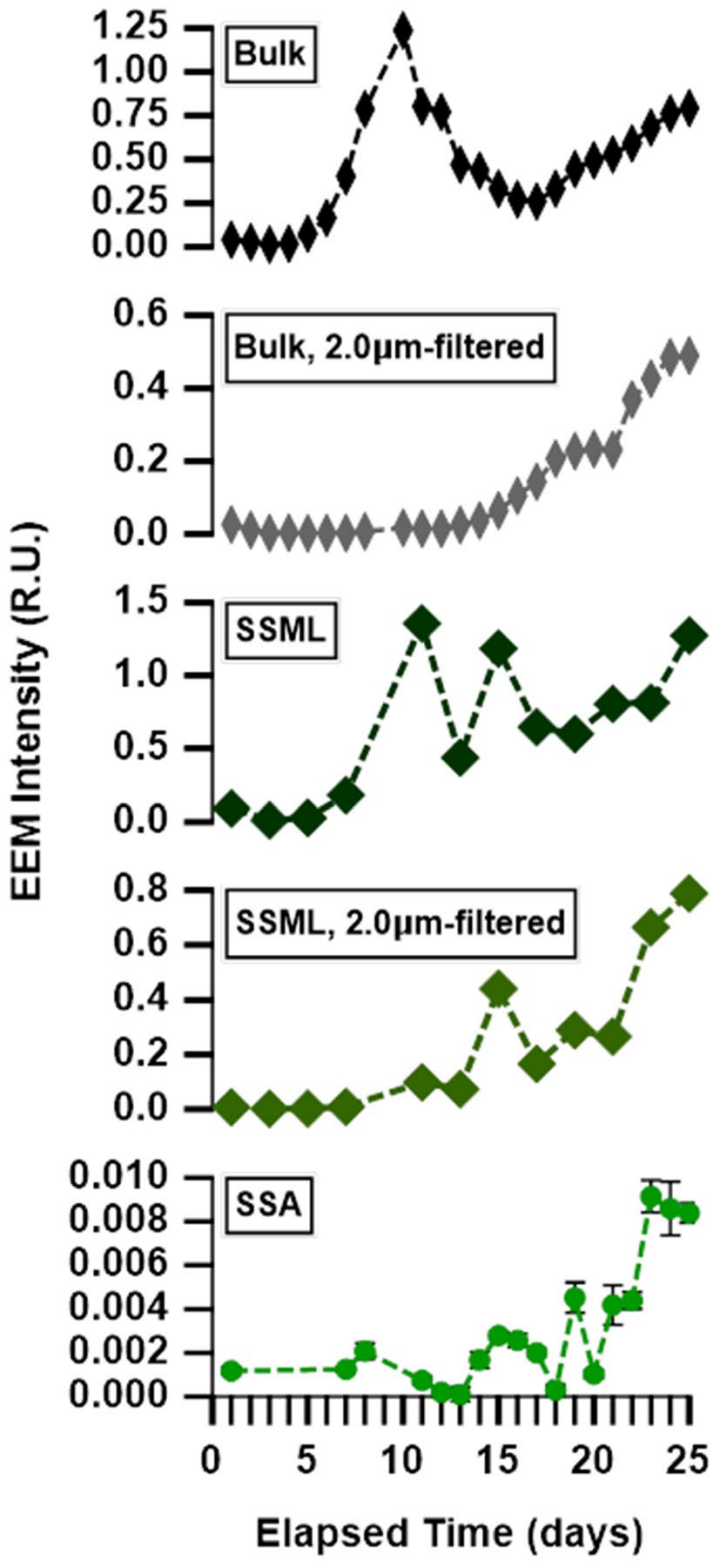
Temporal trends in chlorophyll-a signal from MART B for (top to bottom): unfiltered bulk seawater, bulk seawater after 2.0 μm filtration, unfiltered SSML, SSML after 2.0 μm filtration, and SSA. The signals are quantified in Raman units (R.U., see “Methods”).

Chlorophyll-a fluorescence, observed in MART B EEMs, appeared in all phases (bulk seawater, SSML, and SSA) (Fig. 1f–h). To study the transfer of phytoplankton and bacteria cells to SSA, we examined chlorophyll-a fluorescence in three different seawater size fractions: unfiltered seawater, 2.0 μm-filtered seawater, and 0.2 μm-filtered (Fig. 3). The 2.0 μm-filter removes large phytoplankton and retains bacteria and dissolved organics, while the 0.2 μm-filter removes bacteria and retains dissolved organics in the seawater. Chlorophyll-a concentrations in bulk seawater and SSML showed distinct temporal trends for each of these size fractions. In the unfiltered bulk seawater, the temporal trend in chlorophyll-a for EEMs in MART B tracked the growth and decline of phytoplankton, similar to the hand-held fluorometer (Fig. 1e). After filtering bulk seawater with a 2.0 μm filter to remove large phytoplankton, chlorophyll-a was still present in bulk seawater (Fig. 3) but showed no growth or decay over days 5–15. The presence of chlorophyll-a after 2.0 μm-filtration is indicative of either the presence of chlorophyll-containing cell fragments and organelles or the presence of cyanobacteria, specifically *Prochlorococcus*, which is the most abundant photosynthetic organism in the ocean^50^. Additionally, chlorophyll-a fluorescence was completely removed after filtering bulk seawater and SSML with a 0.2 μm filter (not pictured), which indicates that freely dissolved chlorophyll-a was not present.

The SSA EEMs indicate that the chlorophyll-a increased gradually through the second growth stage, similar to the trends found for the 2.0 μm-filtered bulk seawater and SSML (Fig. 3). The fluorescence trends for the SSA correlate with the trends for 2.0 μm-filtered SSML (r^2^ = 0.86, p < 0.01). The correlation between chlorophyll-a signals of SSA and 2.0-μm filtered bulk seawater is only slightly lower (r^2^ = 0.76, p < 0.01). Therefore, we conclude that the chlorophyll-a in SSA originates from material residing in the 0.2–2.0 micron size range (either in the bulk seawater or SSML), and thus is most sensitive to the transfer of organelles and small cells such as bacteria^39,51,52^. In other words, the transfer of chlorophyll-a from seawater to SSA is likely dominated by cyanobacteria or by cell fragments produced by microbial degradation of whole phytoplankton cells.

### Protein-like excitation–emission matrix components

In MART A and MART B, the protein-like regions, either tryptophan- or tyrosine-like features, or both, were observed in the EEMs of all phases of the blooms (Fig. 1b–d,f–h). Previous studies have shown that the tryptophan-like and tyrosine-like fluorescence signals can be linked to a variety of proteinaceous components, including cell or cell fragments, exopolymeric substances, soluble amino acids/peptides/proteins, and other indolic and phenolic compounds^29,45,53,54^. EEMS of unfiltered bulk seawater and SSML in MART A and B produced tryptophan-like emission signals at ~ 330 nm that overwhelmed the tyrosine-like emission signals at ~ 300 nm (Fig. 1). This trend is expected because EEMs for these phases are similar to signals in the protein region reported previously for marine microorganisms^53^, and tryptophan is generally the strongest emitter from proteins and microbes upon 280 nm excitation^53,54^.

**Figure 4:**
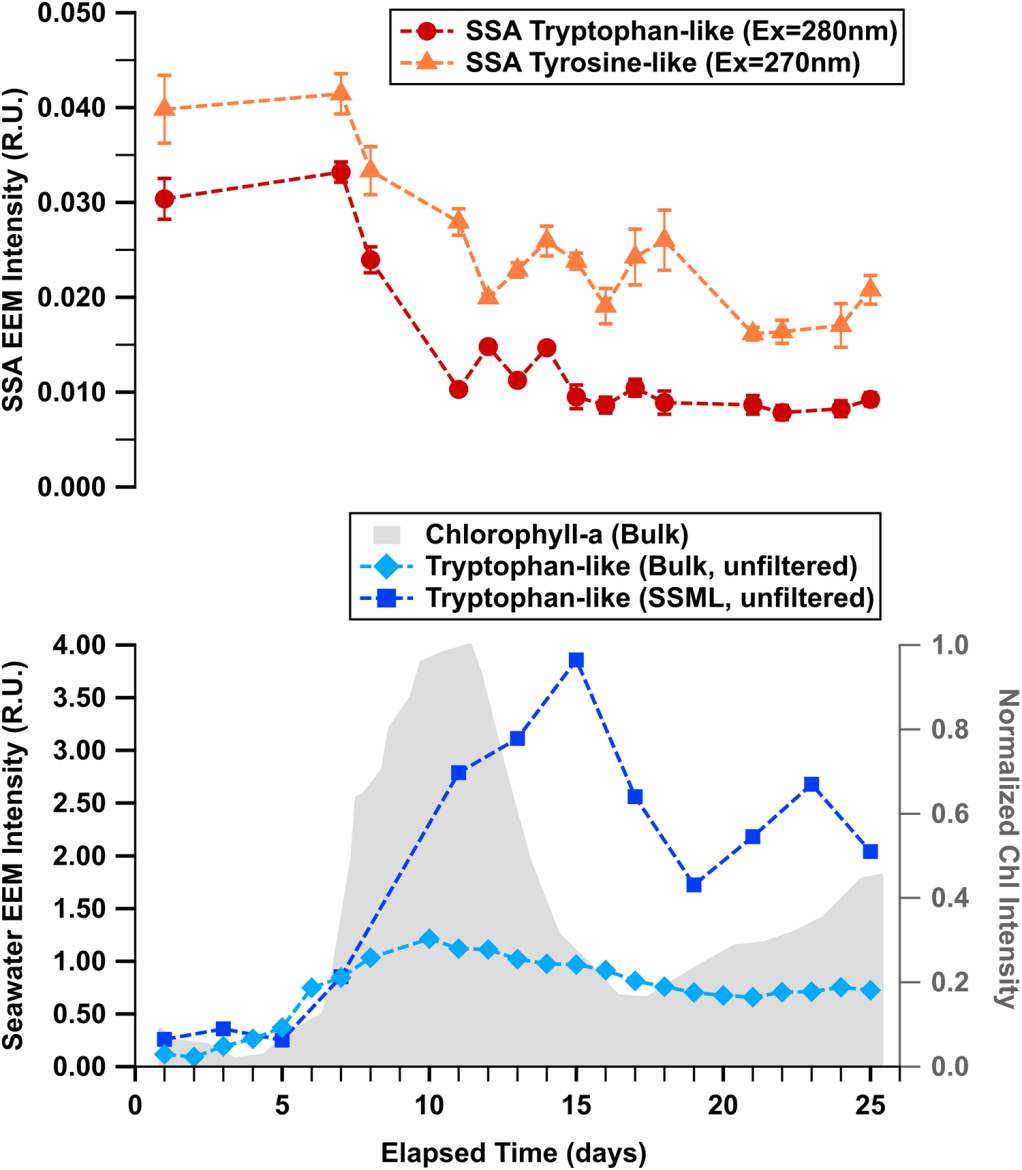
Fluorescence intensity temporal trends in MART B for chlorophyll-a in unfiltered bulk seawater (grey), tryptophan-like substances in bulk seawater and SSML (blue), tryptophan-like substances in SSA (Ex = 280 nm; red circles), and tyrosine-like substances in SSA (Ex = 270 nm; triangles). Error bars represent the standard deviation of the mean (*N* = 9; see “Methods”).

In both MART A and B, the bulk seawater and SSML EEMs showed the greatest tryptophan-like fluorescence intensity either at the peak or the decline stage of the bloom, consistent with the growth of microbes during a phytoplankton bloom (Fig. 4, Fig. S4)^55^. A more surprising trend emerges when examining the protein-like signals in SSA. In contrast to seawater, protein-like fluorescence in SSA for MART B was most intense before the peak of the phytoplankton bloom and decreased over the course of the bloom (Fig. 4). Additionally, EEMs of SSA for both MART A and B primarily showed tyrosine-like fluorescence signal with less contribution from tryptophan-like fluorescence that was dominant in the bulk seawater and SSML phases (Fig. 4, Fig. S4).

The overall decrease in protein-like signal over time and enhanced tyrosine signal in SSA suggests that the principal sources of protein-like fluorescence in the aerosol phase differ from those in bulk seawater/SSML phases^56^. The seawater EEMs showed mainly tryptophan-like fluorescence. As mentioned previously, tryptophan is the dominant fluorophore for microorganisms including bacteria^53^. Bacteria are abundant in seawater, even under non-bloom conditions^57^. Previous studies have observed bacteria in isolated SSA^4^ and a similar peak in microbial signatures in SSA prior to phytoplankton bloom growth^58^.

While the tryptophan-like fluorescence in the bulk seawater and SSML could be attributed to bacteria, the enhanced tyrosine-like fluorescence could be attributed to soluble exudates or extracellular polymeric substances (EPS). EPS makes up a significant fraction of dissolved organic matter in seawater^59,60^. Previous work has shown that tyrosine can dominate EEMs of soluble EPS in some conditions^61^. Thus, we suggest that while bacteria cells are transferred to SSA, sea–air transfer is dominated by soluble exudates. In this scenario, the decrease in protein-like signal in SSA could be due to the colonization of bacteria with particulate matter in seawater, which has been shown to occur in seawater after a phytoplankton bloom^62^, and/or less efficient transfer of soluble exudates/EPS as particles in seawater become stickier and increase in size over the course of a bloom^55,56,60,63^.

### Transfer of humic-like substances from seawater to SSA

HULIS peaks were present in bulk seawater and SSML for both blooms (Fig. 1b,c,f–h). EEMs of bulk seawater in the MART A experiment had an additional HULIS band at 420 nm excitation/490 nm emission (Fig. 1b). The same humic-like peaks present in seawater of MART A were also present in the EEMs of the corresponding SSA (while not apparent in Fig. 1d, these peaks appear on a magnified scale, Fig. S2), while only the 360 nm excitation HULIS peak was present in the SSA EEMs for MART B.

**Figure 5:**
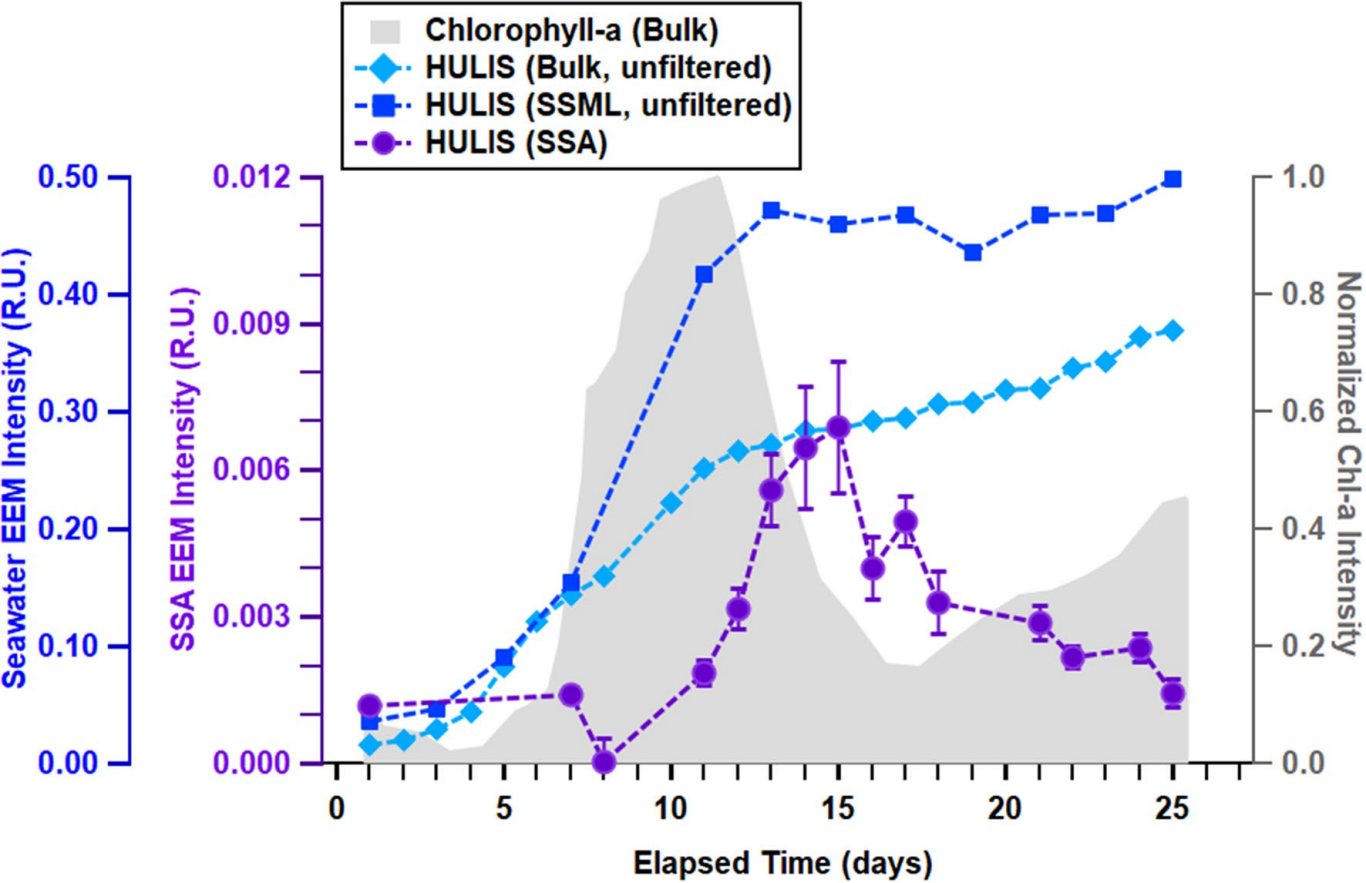
Temporal trends in fluorescence intensity of chlorophyll-a in unfiltered bulk seawater (grey shading), HULIS in unfiltered bulk seawater and SSML (Ex/Em = 360 nm/450 nm; blue diamonds and squares, respectively), and SSA (Ex/Em = 360 nm/472 nm; purple circles). Error bars represent the standard deviation of the mean (N = 9; see “Methods”).

Temporal trends for HULIS fluorescence in the SSML of MART B showed a large increase roughly coincident with the chlorophyll-a growth, followed by a leveling off as the bloom progresses and declines (Fig. 5, squares). In this same MART, bulk seawater showed an initial rise in HULIS fluorescence coincident with that of the SSML, then a gradual increase after the bloom peak (Fig. 5, diamonds). In contrast to seawater, the HULIS fluorescence of the SSA (Fig. 5, circles) reached its maximum *after* the peak in chlorophyll-a in bulk seawater, and eventually decreased to initial levels. The MART A microcosm showed similarly contrasting trends for HULIS fluorescence of the SSML and SSA phases. Separate from the temporal trends, we note a slight (~ 20 nm) redshift in the fluorescence spectra from the start of the bloom to the peak stage, followed by a return to the original wavelength late in the bloom (Fig. S5).

The delay in the increase of SSA HULIS fluorescence has not been reported previously. This delay is a clear indication that phytoplankton in bulk seawater are not direct drivers of HULIS transfer from the ocean to the atmosphere. Additionally, this lag between the peaks in chlorophyll-a and SSA HULIS in SSA is consistent with the increase in bacteria concentrations typically observed in seawater after the peak of a phytoplankton bloom^5,15,16,64,65^. Bacterial degradation is vital for HULIS production and HULIS chemical composition^30,66^. This connection with bacteria suggests, as previous studies have shown, that microbial interactions play a critical role in the enrichment of organic matter, particularly HULIS, in SSA^5,15,16^.

The decline in SSA HULIS emission despite the increase or plateau in bulk seawater and SSML, respectively, can be explained by changes in the chemical nature of the HULIS. The spectral changes in SSML HULIS (~ 20 nm redshift), which nearly coincided with the increase and subsequent decrease of HULIS fluorescence in the SSA, suggest that bacteria induce changes in HULIS chemical composition over the course of a bloom^67,68^. For example, there may be a shift to a more surface-active form of HULIS, which would impact the efficiency of HULIS transfer from sea surface to SSA. While further chemical and size characterization studies are required to identify the mechanism for HULIS transfer, these findings clearly demonstrate that the HULIS concentration in seawater alone does not correlate with the transfer and peak HULIS concentration in SSA.

### Implications for sea-to-air transfer of organic matter

Identifying the factors that control the release of biomolecules and microbes from the ocean to the atmosphere is critical because these biogenic organic species have different influences on clouds, climate, weather, and atmospheric chemistry^63,69–71^. For example, microbes identified using protein fluorescence are likely contributors to ice formation in clouds^70,71^, while humic-like substances have been linked to cloud nucleation and atmospheric photochemistry, such as the production of reactive gases like nitrous acid in the atmosphere^72,73^. Here, we used a lab-controlled microcosm in conjunction with rapid analysis via EEM spectroscopy to monitor changes and probe connections in the bulk seawater, SSML, and SSA. While previous studies have attempted to correlate SSA organic enrichment with parameters such as SSA particle flux or seawater DOC concentrations, we found no links between changes in fluorescent signatures and SSA particle flux (number, surface area, or volume) or DOC concentrations (Fig. S6)^18,19^. Instead, our study reveals three major findings regarding sea–air transfer of organic matter:

First, none of the classes of organics investigated (chlorophyll-a, protein-like substances, and HULIS) showed a dependence on the growth of phytoplankton as indicated by chlorophyll-a in the bulk seawater. This suggests that phytoplankton are not producing the majority of the fluorescent organic matter that is directly transferred to SSA. Instead, phytoplankton likely play a key role in producing the organic precursors to those species that ultimately become transferred to SSA. This also suggests that microbial degradation controls the transfer of organic matter, breaking down constituents into the appropriate size range and proper chemical properties for efficient sea–air transfer^5,9,15,16,20^.

Second, we show that the peaks in SSA of different organic species do not correspond to peaks for these same species in bulk seawater or the SSML. Thus, this study reveals that factors beyond bulk seawater and SSML concentrations control the concentrations of biogenic species in SSA in the atmosphere. Previous studies have shown selective transfer occurs, for example due to enhanced surface adsorption^21^ or microbe cell membrane chemistry^74^. This study suggests that processes that can alter the size and chemical properties of biogenic organic matter play an important role in controlling sea–air transfer.

**Figure 6:**
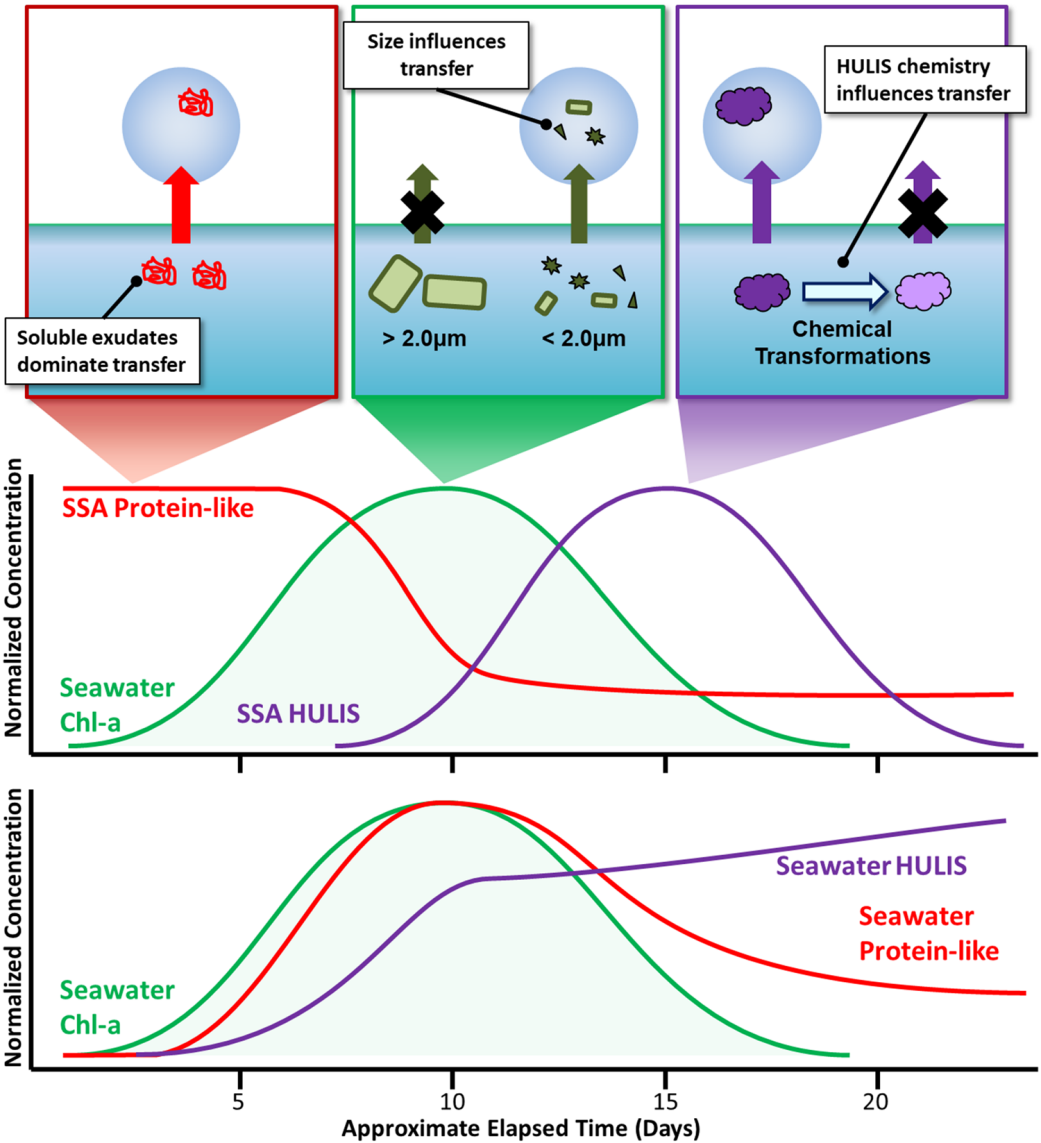
Summary of EEM chromophores (chlorophyll-a, humic substances, and protein-like) and factors that are likely to have an influence on transfer to aerosol particles. Approximate concentrations are normalized to their respective maxima.

Finally, transfer of the three different organic classes studied here occurred at different points during the bloom, suggesting different factors controlled the release of each species (Fig. 6). The transfer of each class was likely affected by different physical, biological, and chemical factors that depend on microbial conversion processes to modify size and chemical properties^16^. Chlorophyll-a transfer requires the *breakdown* of cells into cell fragments and organelles (Fig. 6)^51,52^. The transfer of protein-like substances is reduced by *colonization* of particulate matter by bacteria likely linked to the formation of “sticky” exudates (Fig. 6). The transfer of HULIS depends on *chemical* changes induced by bacteria (Fig. 6). The transfer of these classes into SSA at different times clearly shows that while SSA can be enriched with organic species, the specific types of organic molecules in SSA change over time, which will lead to different chemical and climate-relevant properties such as water uptake, cloud formation, and ice nucleation.

By isolating the ocean and atmosphere during a bloom, these studies are the first to begin unraveling the mechanisms by which ocean-derived biological species in seawater are transferred into SSA. Future experiments will be performed to probe how changes in seawater particulate size distributions, colonization, marine gel production, and other ocean processes affect the transfer of dissolved and particulate organic matter from the ocean to the atmosphere. The ultimate goal will be to better understand the factors controlling the biogeochemical cycling of organic species between the ocean and atmosphere.

## Supplementary Information

Supplementary Information.

## Data Availability

The datasets generated during and/or analyzed during the current study are available at: 10.6075/J0NC5ZQ1.
