# Supplementary material for: Factors controlling the transfer of biogenic organic species from seawater to sea spray aerosol
Source: Sci Rep. 2022 Mar 4;12:3580. doi: 10.1038/s41598-022-07335-9 (PMC8897391; doi:10.1038/s41598-022-07335-9)
Supplement: Supplementary file 1 — Supplementary Information. [file 41598_2022_7335_MOESM1_ESM.pdf]

Supplementary Information

Factors controlling the transfer of biogenic organic species from seawater to sea spray aerosol

Mitchell V. Santander[1], Jamie M. Schiffer[2], Christopher Lee[3], Jessica L. Axson[4], Michael J. Tauber,[1] and Kimberly A. Prather[1,3]*

[1] Department of Chemistry and Biochemistry, University of California, San Diego, La Jolla, CA 92093, U.S.A.
[2] Takeda, San Diego, CA 92121, U.S.A.
[3] Scripps Institution of Oceanography, University of California, San Diego, La Jolla, CA 92037, U.S.A.
[4] Independent Scholar
*To whom correspondence should be addressed: Kimberly Prather (kprather@ucsd.edu)

Contents	Description	Page
**Figure S1**	Example EEM depicting fluorphore regions	2
**Figure S2**	Selected EEMs	3
**Figure S3**	Selected spectra for HULIS and protein-like components	4
**Figure S4**	Trends in EEMs amplitudes for protein-like components	5
**Figure S5**	Selected spectra of HULIS in SSML phase	6
**Figure S6**	Relationship of fluorescence, DOC, and APS size distributions	7
**Table S1**	PARAFAC components and descriptions	8
**Table S2**	Seawater collection times and conditions	8

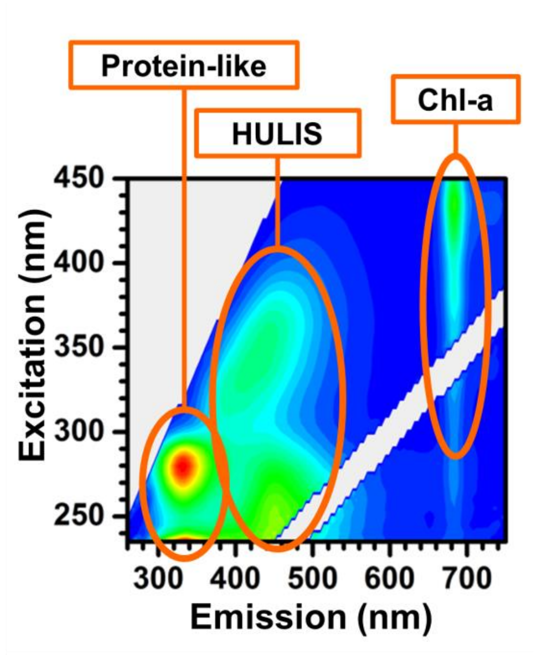

Figure S1. Example EEM depicting the locations of seawater fluorophore regions

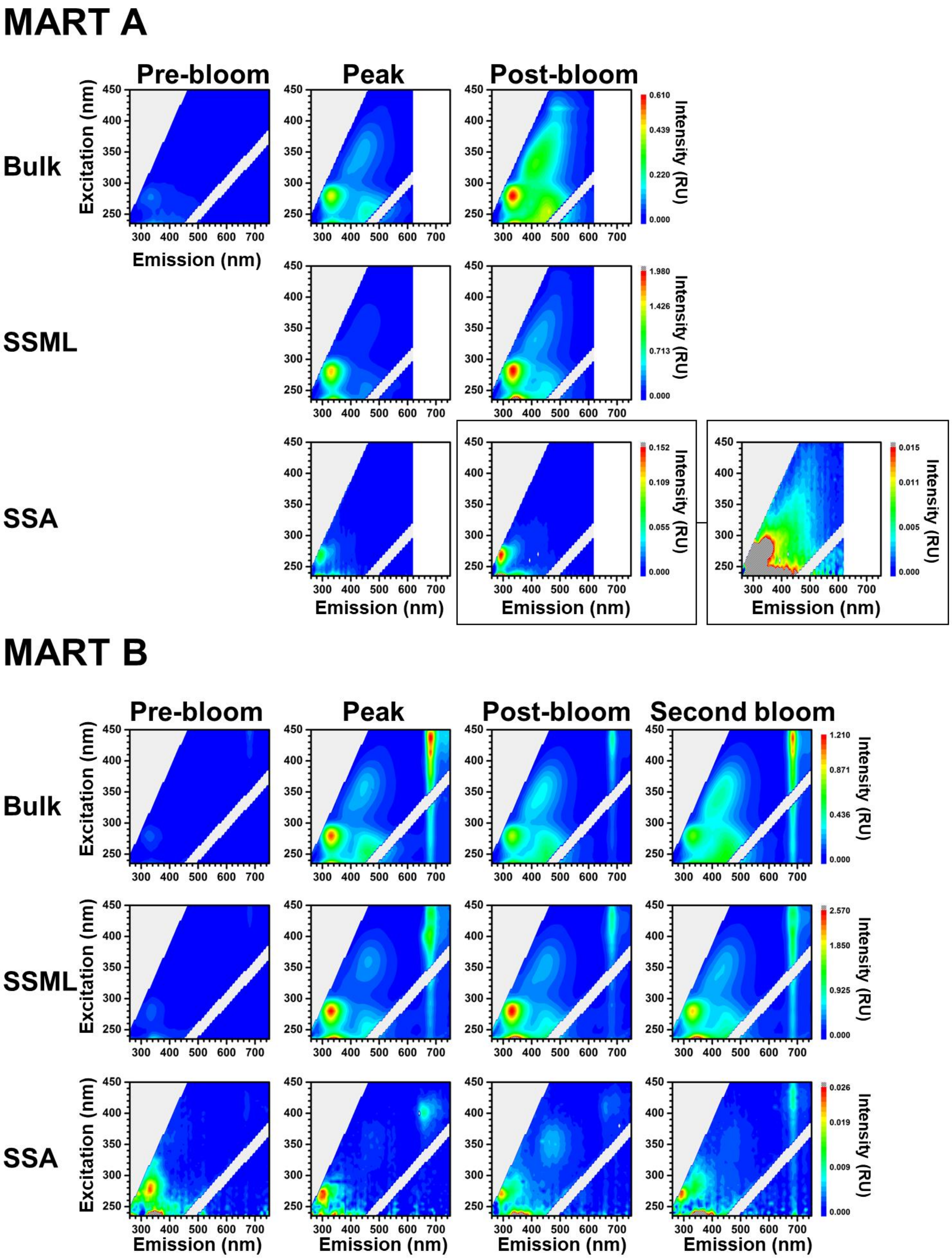

Figure S2. Selected EEMs for bulk seawater, SSML, and SSA at different stages for MART A and MART B mesocosms. The EEM for MART A post-bloom SSA is shown with two different scales to show the presence of the humic-like fluorescence bands.

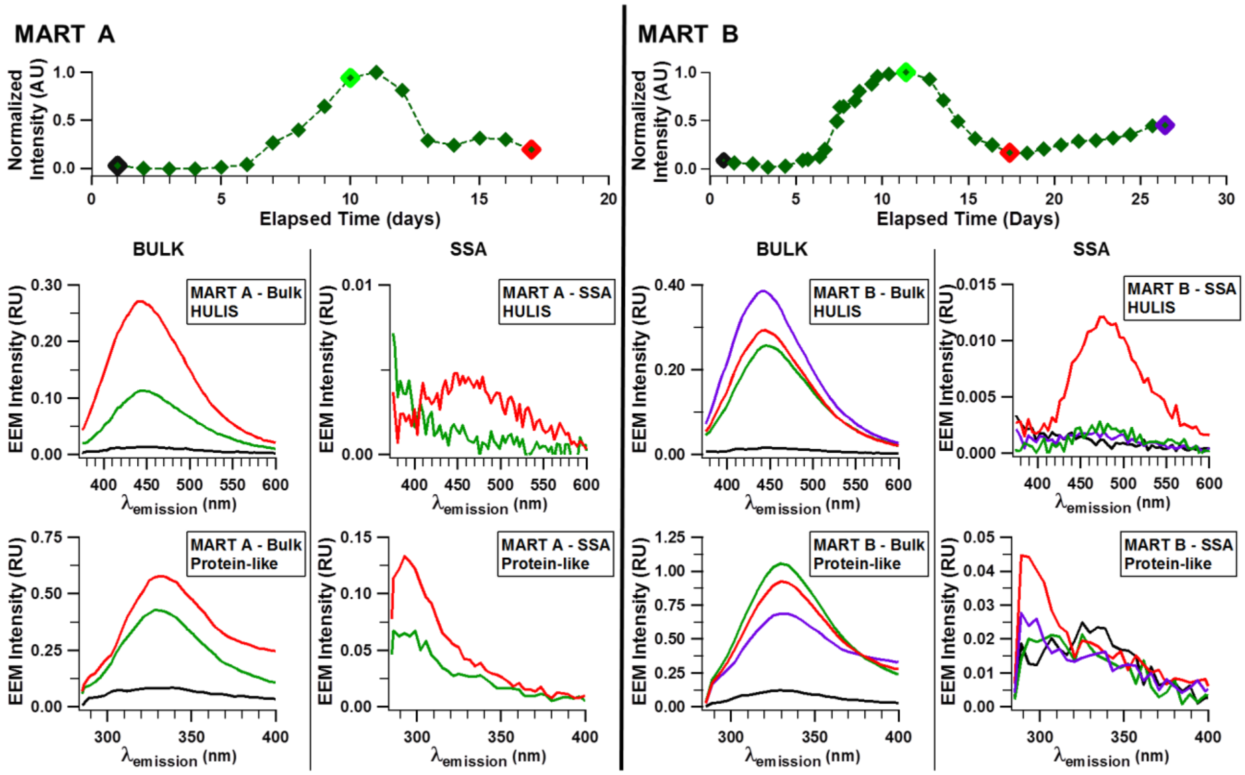

Figure S3. MART A and MART B chlorophyll-a temporal trends (top) and select emission spectra for bulk seawater and SSA. Panels labeled "HULIS" or "Protein-like" were excited with 360 nm or 275 nm light, respectively. Colors correspond to different stages: pre-bloom (black), bloom peak (green), post-bloom (red), and second bloom (purple).

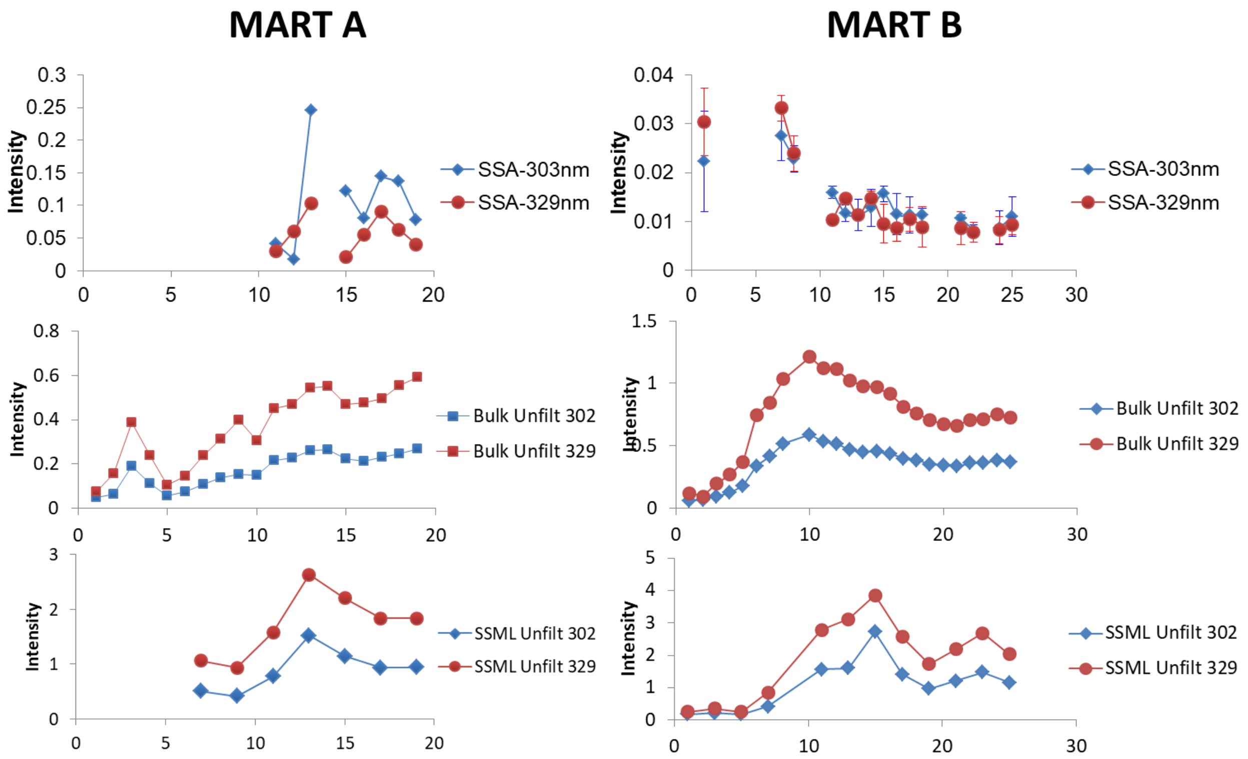

Figure S4. Trends in the EEMs amplitudes, at wavelength positions that report on the tyrosine-like component (Ex/Em = 280 nm / 303 or 305 nm) and tryptophan-like component (Ex/Em = 280 nm / 329nm). The amplitudes (intensity) are taken directly from the EEMs, and are not from PARAFAC analysis. The first row shows trends in the SSA phase. MART A samples were collected only once. MART B samples were collected three times, and error bars show one standard deviation. The gaps in data reflect samples with unusually high or low fluorescence relative to the other samples and are excluded as outliers. The second and third rows show trends for the unfiltered bulk and SSML

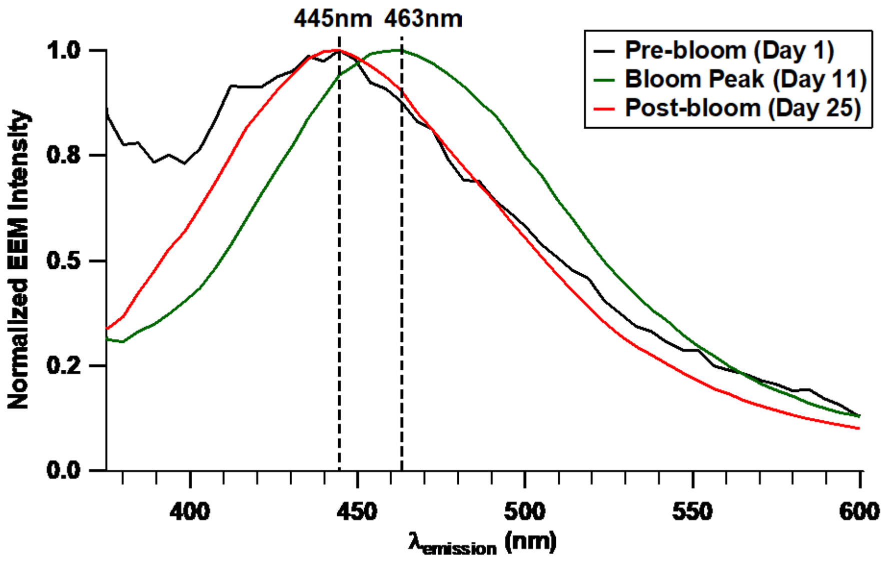

Figure S5. Emission spectra for SSML samples of MART B, excited at 360 nm to probe the HULIS component.

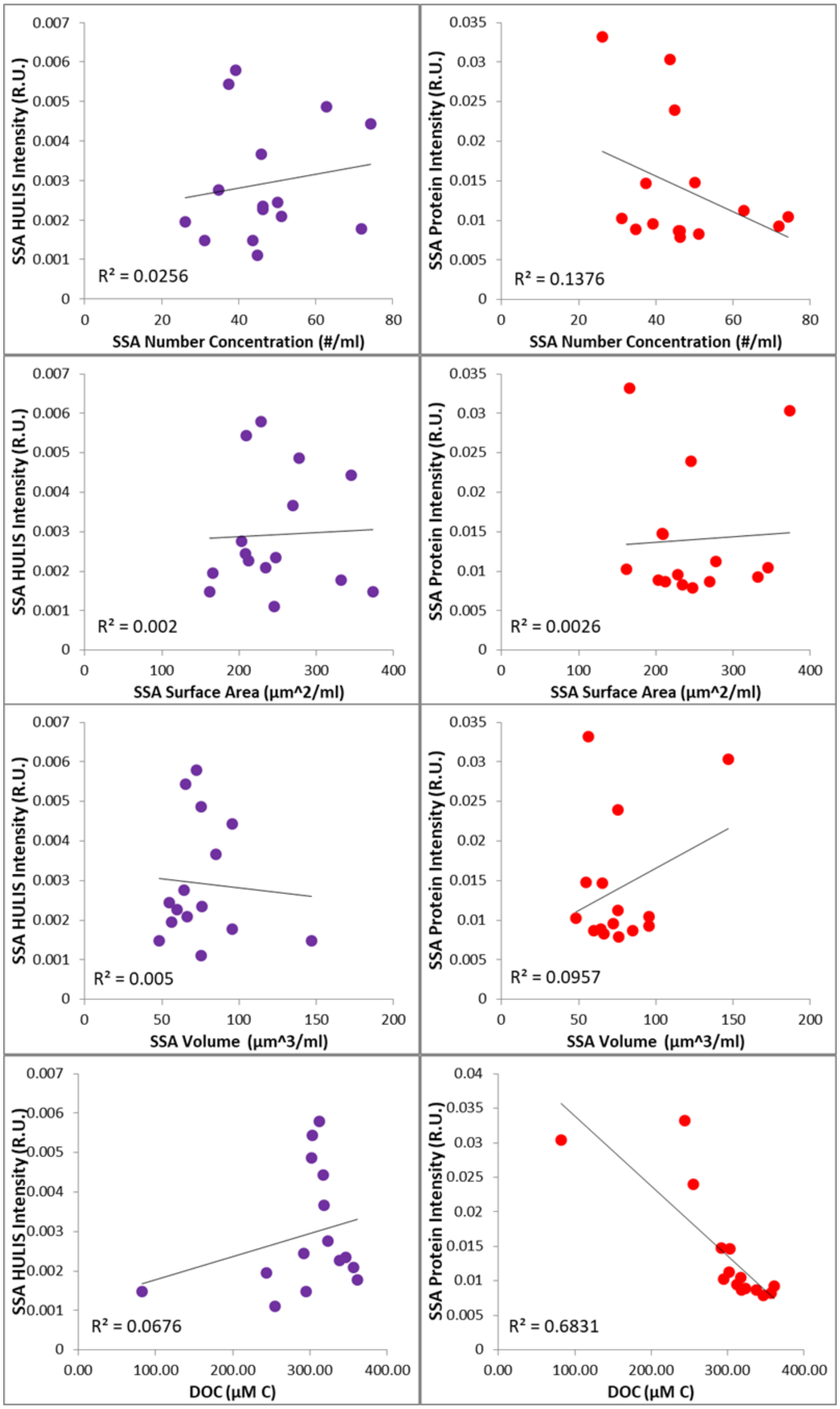

Figure S6. Relationships of HULIS (left) and protein-like substances (right) versus SSA particle number, surface area, and volume concentrations, and DOC.

Table S1. Excitation and emission maxima for each of the PARAFAC components identified for MART A and MART B

Component	***Ex (nm)***	***Em (nm)***	***Description***
**MART A**			
**A1**	280	330	Tryptophan-like
**A2**	255, 365	453	Humic
**A3**	325	396	Humic
**A4**	<245, 275	340	Tryptophan-like
**A5**	270	302	Tyrosine-like
**A6**	275, 420	492	Humic
**MART B**			
**B1**	280	330	Protein-like, tryptophan-like
**B2**	255, 365	460	Humic
**B3**	435	684	Chlorophyll *a*
**B4**	<245, 325	394	Humic

Table S2. Seawater collection times and conditions for MART A and B

Date	***Chlorophyll-a (mg m^{-3})***	***Water Temp. (^{o}C)***	***Pressure (dbar)***	***Salinity (PSU)***	***MART***
**1/15/2014 20:00**	1.33	15.30	4.83	33.54	A
**4/11/2014 12:00**	4.49	14.34	3.51	33.43	B
